# Proteomic Study of Diffuse Large B-Cell Lymphoma Identifying Proteins Associated with R-CHOP Response

**DOI:** 10.3390/biomedicines13112709

**Published:** 2025-11-04

**Authors:** Hulda Haraldsdóttir, Rasmus Froberg Brøndum, Marie Hairing Enemark, Bent Honoré, Maja Ludvigsen, Christopher Aboo, Allan Stensballe, Judit Mészáros Jørgensen, Hanne Due, Karen Dybkær

**Affiliations:** 1 Department of Hematology, Aalborg University Hospital, 9000 Aalborg, Denmark; h.haraldsdottir@rn.dk (H.H.); hanne.rasmussen@rn.dk (H.D.); 2 Department of Clinical Medicine, Aalborg University, 9260 Gistrup, Denmark; 3 Clinical Cancer Research Center, Aalborg University Hospital, 9000 Aalborg, Denmark; as@hst.aau.dk; 4 Center for Clinical Data Science, Aalborg University Hospital, and Aalborg University, 9260 Gistrup, Denmark; rfb@rn.dk; 5 Department of Hematology, Aarhus University Hospital, 8000 Aarhus, Denmark; mariem@rm.dk (M.H.E.); majlud@rm.dk (M.L.); judit.joergensen@aarhus.rm.dk (J.M.J.); 6 Department of Clinical Medicine, Aarhus University, 8000 Aarhus, Denmark; 7 Department of Biomedicine, Aarhus University, 8000 Aarhus, Denmark; bh@biomed.au.dk; 8 Department of Health Science and Technology, Aalborg University, 9000 Aalborg, Denmark

**Keywords:** diffuse large B-cell lymphoma, R-CHOP, proteomics, prognostic markers

## Abstract

**Background/Objectives:** Diffuse large B-cell lymphoma (DLBCL) is a molecularly and pathogenically heterogenous disease with varying clinical outcomes, as reflected by the significant number of patients who develop relapse/refractory disease (rrDLBCL) following standard treatment with the combined R-CHOP regimen. The molecular background of rrDLBCL is not yet fully understood, and prognostic and/or companion diagnostic biomarkers for identification and treatment stratification of these patients are in high demand. **Methods**: This exploratory study used comprehensive proteomic data to identify proteins associated with treatment response. Proteome profiles of DLBCL cells were analyzed through groupwise comparison between cell lines with a resistant or sensitive response to rituximab, cyclophosphamide, doxorubicin, and vincristine. Their responses were determined using subsequent drug response screens, mimicking the conditions of diagnostic samples prior to treatment. **Results**: A total of 98 differentially abundant proteins, including NSFL1C, GET4, PCNA, and SMC5, were found between resistant and sensitive cells. These same 98 proteins were examined in two cohorts of DLBCL patients, leading to the identification of 16 proteins whose expression was consistently associated with treatment response both in vitro and in patient tissue samples. Among these, GET4 and NSFL1C showed the highest enrichment in R-CHOP resistant patients compared to sensitive responders. In the cell line study, GET4 was enriched in cyclophosphamide-resistant cell lines and NSFL1C enriched in vincristine-resistant cell lines, associating GET4 and NSFL1C enrichment in patient samples to responsiveness to cyclophosphamide and vincristine, respectively. Enrichment of DNA damage repair proteins was observed within the differential proteins, highlighting the need to investigate DNA damage repair involvement in treatment responses. **Conclusions**: This study identifies 16 proteins with concordant treatment response specificity in DLBCL cell lines and lymphoma tissue patient samples, suggesting their potential as prognostic markers for DLBCL.

## 1. Introduction

Diffuse large B-cell lymphoma (DLBCL) is an aggressive non-Hodgkin lymphoma entity originating from B lymphocytes. It is the most common type of lymphoma among adults, with an age-adjusted incidence rate of 5.6 per 100,000 each year in the U.S. [1]. DLBCL is a molecularly heterogeneous disease with varying treatment efficacies and clinical outcomes [2,3]. The current first-line standard of care is the immunochemotherapy regimen R-CHOP, consisting of rituximab (R), cyclophosphamide (C), doxorubicin hydrochloride (H), vincristine sulfate (O), and prednisone (P) [3]. Rituximab is a CD20-targeting monoclonal antibody that induces complement-mediated and antibody-dependent cell cytotoxicity and apoptosis [4]. Cyclophosphamide and doxorubicin are DNA-damaging drugs that act through alkylating and topoisomerase II-inhibiting effects [5], respectively. Vincristine is an anti-microtubule drug, inhibiting mitosis, while prednisone is a steroid [5]. Despite this regimen of effective antitumor drugs, approximately 40% of patients receiving the combination treatment are not cured but develop refractory disease or relapse (rrDLBCL) [3,6]. The salvage treatment options for rrDLBCL patients include chimeric antigen receptor (CAR) T-cell therapy or platinum-based treatment regimens, followed by autologous stem cell transplantation (ASCT) [7,8]. However, many rrDLBCL patients are non-tolerant for intensive salvage therapy due to toxicities, and a substantial fraction of patients are ineligible for ASCT due to comorbidities, performance score, or older age [9,10,11]. Moreover, approximately 50% of patients who undergo transplantation subsequently relapse [9,12,13]. Therefore, better care options are needed, highlighting the importance of understanding resistance development and its potential for identifying prognostic markers. The molecular heterogeneity of DLBCL has led to the characterization of several distinct molecular subgroups based on genetic and transcriptional profiles of the tumor, where the subgroups differ in affected oncogenic signaling pathways and survival rates of patients [14,15,16,17,18]. In an attempt to improve clinical outcomes, subgroup-specific treatment approaches have been tested in clinical trials; however, only minor improvements have been achieved, and R-CHOP remains the standard of care [19,20,21,22,23,24]. Therefore, new approaches to stratify the molecular heterogeneous group of DLBCL patients are needed. For this purpose, proteomic studies of DLBCL that characterize the effector molecules within the cells have received increasing attention [25,26,27]. With large-scale identification of differentially abundant proteins in DLBCL cells, markers for treatment response can be identified, enabling indirect insights into the molecular mechanisms controlling the response. Proteomic profiles can help identify patients with a higher risk of relapse, i.e., those needing additional and continuous care or follow-up in the clinic. In this study, we use comprehensive proteomic relative quantitative data from DLBCL cell lines to improve our understanding of how the proteomic background of DLBCL affects preparedness for treatment response. This study aims to identify proteins associated with treatment response through differential analysis of protein profiles of baseline DLBCL cell lines, which are subsequently characterized as resistant or sensitive to single-drug components of R-CHO.

## 2. Materials and Methods

### 2.1. Cell Lines

A total of 16 DLBCL cell lines were included for proteomic and dose–response analysis, namely DB, FARAGE, HBL-1, HT, MC-116, NU-DHL-1, NU-DUL-1, OCI-Ly19, OCI-Ly3, OCI-Ly7, OCI-Ly8, RIVA, SU-DHL-10, SU-DHL-4, SU-DHL-5, and U-2932. The FARAGE cell line was purchased from the American Type Culture Collection (ATCC, Manassas, VA, USA), HBL-1 was purchased from Applied Biological Materials (abm, Richmond, BC, Canada), and OCI-Ly8 was kindly provided by Hans Messner, Canada. The remaining cell lines were purchased from the German Collection of Microorganisms and Cell Cultures (DSMZ, Braunschweig, Germany). Cells were cultured at 37 °C and 5% CO_2_ in RPMI-1640 + 10–20% Fetal Bovine Serum and 1% penicillin–streptomycin (Supplementary Table S1). They were passaged twice a week, authenticated by DNA barcoding, and examined for mycoplasma during the study.

### 2.2. Protein Analysis

#### 2.2.1. Sample Preparation for Protein Analysis

The cell lines were thawed and gently washed in cold PBS, followed by centrifugation at 300× *g*. The cells were lysed in 5% sodium deoxycholate, aliquoted to 100 µg, measured using OD280, and transferred to YM-10 spin filters (Millipore; Darmstadt, Germany). This was followed by buffer exchange to digestion buffer (5% sodium deoxycholate in 0.05 M TEAB: pH 7.8). The lysates were reduced using 12 mM TCEP at 37 °C for 30 min, and then alkylated using 40 mM iodoacetamide for 30 min. This was followed by two rounds of buffer exchange using centrifugation at 14,400× *g*. Trypsin (1 µg) was added to each sample, which were then incubated overnight. Sodium deoxycholate was precipitated using 5% formic acid, resulting in soluble peptide recovery.

#### 2.2.2. LC-MS/MS Data Acquisition

A sample volume of 5 µL (approx. 15% of protein extract) was injected onto a Dionex Ultimate 3000 nanoLC system connected to a Quadrupole Orbitrap (QExecutive QE+) mass spectrometer equipped with a NanoSpray Flex ion source (Thermo Scientific, Bremen, Germany). Samples were loaded onto the trapping column (Acclaim PepMap100 C18, 5 µm column from Thermo Scientific) at a flow rate of 8 µL per minute. A 50 cm Acclaim Pepmap RSLC 75 µm analytical column, connected with nanoViper fittings, was used for peptide separation at a nanoflow rate of 300 nL per minute. Nano-electrospray was performed using a Picotip ‘Silicatip’ emitter from New Objectives. The LC buffers were buffer A (99.9% water, 0.1% formic acid (FA)) and buffer B (99.9% Acetonitrile (AcN), 0.1% FA). A gradient from 10 to 45% buffer B was applied over 120 min.

#### 2.2.3. Protein Identification and Quantitative Data Analysis

Proteins were identified using MaxQuant (v 1.6.12.0), which analyzed the LC-MS/MS mass spectra data against UniProt (March 2020; human reference proteome with isoforms) using the label-free quantitation (LFQ) algorithm. Standard settings were used, including a peptide and protein false discovery rate of 1%, as well as at least two peptides for protein quantification. Reversed sequences were used as decoys, and contaminant sequences were added automatically by MaxQuant. *p*-values were calculated using a two-tailed, heteroscedastic *t*-test. Quantitative values were calculated as the averages of MaxQuant LFQ values based on at least two values per condition.

#### 2.2.4. Protein Abundance

The relative abundance of proteins measured by LFQ was computed using Perseus (v. 1.6.15.0) software. The data was log_2_-transformed and filtered for reverse hits, proteins identified only by site, and potential contaminants. Only proteins detected in more than 50% of all samples were included for analysis. Missing values were imputed (0.3–1.8) from a normal distribution using default settings. Normalization of protein abundance, including imputed values, was ensured for each sample. For pairwise comparison of the protein abundance duplicates from each cell line, the Pearson coefficient correlation was estimated (0.57–0.89). Protein abundances were summarized using the median value. After quality checking and filtering, 4716 proteins remained for analysis. The mass spectrometry proteomics cell line data have been deposited in the ProteomeXchange Consortium via the PRIDE [28] partner repository with the dataset identifier PXD064483.

### 2.3. Dose–Response Experiments

Rituximab, doxorubicin, and vincristine were obtained from Aalborg University Hospital, Denmark. Since cyclophosphamide requires hepatic activation to generate its active metabolite, the synthetic oxazaphosphorine derivative, mafosfamide was used as a surrogate in the dose–response analysis, purchased from Niomech, Germany (CAT#D-18864). The effect of rituximab, mafosfamide, doxorubicin and vincristine on viable proliferating cells was analyzed by performing systematic dose–response experiments as described by Falgreen et al. [29]. These dose response analyses are based on unbiased exposure time independent summary statistics, meaning that varying cell line growth kinetics, variation in seeding concentration, and drug exposure time are accounted for. Briefly, cells were seeded in 96-well plates (Cat#655 180, Greiner CELLSTAR) according to a cell line-specific density (Supplementary Table S1). Drugs were added 24h post-seeding in triplicates for 18 or 16 increasing concentrations, prepared as twofold dilution series. The highest drug concentrations were 133.3, 80, 10, and 20 µg/mL for rituximab, mafosfamide, doxorubicin and vincristine, respectively (Supplementary Table S2). Three to six wells were used as untreated controls and other three to six wells as background controls. For rituximab drug screens, activated human serum was added to the cell culture 30 min after drug addition to mirror the complement-mediated cytotoxicity of rituximab. After 48h of drug exposure, cell viability was estimated from triplicate absorbance measurements with an MTS assay at 492 nm, using the Fluostar Optima (BMG LABTECH, Ortenberg, Germany) with CellTiter reagent (CellTiter 96 Aqueous One Solution Reagent, Promega, Madison, WI, USA). Doxorubicin is a colored agent, and therefore, correction for background absorbance was performed using the methods of Falgreen et al. [29]. The cell viability estimation was repeated up to 12 times for biological replicates. Dose–response curves were created for the cell lines and the area under the positive part of the curves (AUC) was used as the summary statistics. The generated AUC value for each cell line for the respective drugs can be seen in Supplementary Table S3. For each drug, cell lines were ranked based on their AUC values, with the highest 40% classified as resistant and the lowest 40% as sensitive. The remaining 20% were designated as intermediate.

### 2.4. Clinical Cohorts

Proteomic studies of clinical cohorts by Fornecker et al. [30] and Ludvigsen et al. [31] were used to assess the clinical relevance of the differentially abundant proteins identified in resistant and sensitive cell lines in this study. Both cohorts included diagnostic DLBCL patients with a known R-CHOP response. Fornecker et al. included 20 fresh-frozen tissue samples collected at diagnosis before the patients received any treatment. After uniform treatment with R-CHOP, patients with stable or progressive disease and those who relapsed within one year after complete response were classified as chemorefractory, referred to here as chemoresistant (*n* = 8). Patients with complete response and no relapse within 24 months after treatment were classified as chemosensitive (*n* = 12). In the study by Ludvigsen et al., diagnostic formalin-fixed paraffin-embedded tumor tissue samples (*n* = 64) were included. All patients responded initially to R-CHOP. However, 11 patients relapsed within two years of follow-up, considered as chemoresistant in the present study, whereas the chemosensitive patients did not relapse within two years. To sum up, the tissue samples analyzed originated from 53 chemosensitive and 11 chemoresistant patients. Both cohorts performed label-free quantitative proteomics, detecting 4774 proteins in the Fornecker cohort and 2026 proteins in the Ludvigsen cohort. Differential protein abundance analysis between the chemoresistant and chemosensitive patient groups was performed to identify proteins associated with R-CHOP responses.

The differentially abundant cell line proteins identified in this study, as well as their abundance patterns in chemosensitive and chemoresistant DLBCL patients, were analyzed within the clinical cohorts. The concordance of abundance differences between resistant and sensitive groups in both cell line and patient data was demonstrated for NSFL1C, GET4, PCNA, and SMC5 by plotting their LFQ values.

### 2.5. Statistical Analysis

To compare protein abundances between cell lines that respond differently to the respective drugs within R-CHO, comparison of cell lines representing the two responsive extremes were made, i.e., resistant vs. sensitive, and intermediate excluded. For each drug, the differential analysis of resistant and sensitive cell lines was performed with a two-sample unpaired Student’s *t*-test using the software program Perseus. Proteins with a *p*-value < 0.01 were considered significantly differentially abundant between resistant and sensitive cell line groups, as Benjamin–Hochberg correction showed no proteins with a q-value < 0.05. The significant proteins were further examined with pathway analysis using Metascape (December 2024) [32,33], with the inclusion of Gene Ontology (GO) and reactome pathways (R-HSA). Protein abundance comparisons between resistant and sensitive groups in the cell line and patient data from the Ludvigsen cohort are presented with plotted LFQ values. Significance for differentially abundant cell line proteins is presented with * = *p*-value < 0.01. Hierarchical clustering using Spearman correlation as the distance measure was performed, and the results are presented in a heatmap created using R (4.4.2). Histograms for drug-specific AUC values of the cell lines and enriched terms, as well as violin plots for LFQ values, were created in GraphPad Prism (10.0.0).

## 3. Results

### 3.1. Proteome Profiling and Drug Response of DLBCL Cell Lines

**Figure 1:**
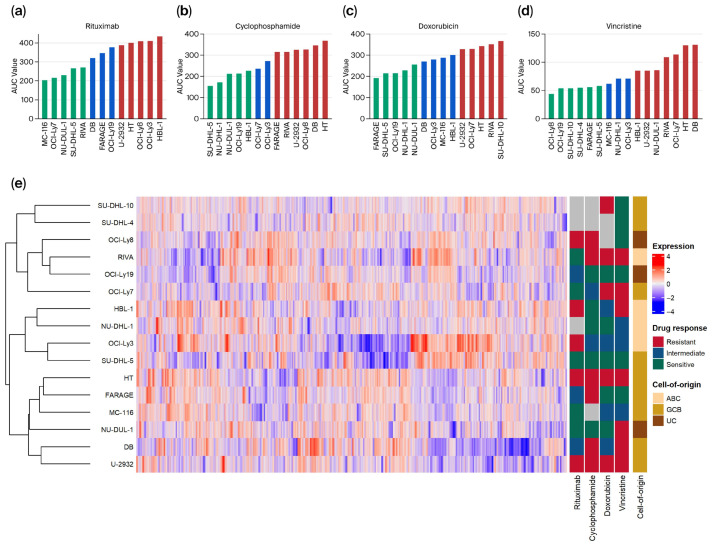
Overview of dose–response and proteomics data from diffuse large B-cell lymphoma (DLBCL) cell lines. (**a**–**d**) For the respective drugs, area under the dose–response curve (AUC) values were used as summary statistics for dose–response screens and plotted for all included cell lines from lowest to highest for each drug. The cell lines with the lowest 40% of AUC values were categorized as sensitive (green), the highest 40% as resistant (red), and the cell lines between as intermediate (blue). (**e**) Hierarchical clustering with a heatmap of protein abundances in the cell lines. Treatment response classification for each drug and cell-of-origin subtype of each cell line is presented on the right. ABC, Activated B-cell-like; GCB, Germinal center B-cell-like; UC, Unclassified.

In this study, 16 untreated DLBCL cell lines were analyzed for baseline proteome profiles using high-throughput quantitative liquid chromatography–mass spectrometry (LC-MS/MS). A total of 4716 proteins were identified and quantified in at least 50% of all samples after filtering for potential contaminants, reverse hits, and proteins only identified by site. On these 16 DLBCL cell lines, systematic dose–response experiments of individual compounds of R-CHO (rituximab, cyclophosphamide, doxorubicin, and vincristine) were subsequently conducted. The dose–response experiments are based on a previously described mathematical G-model for drug induced cell growth inhibition [29] generating dose–response curves for the cell lines corrected for individual cell line growth rate. The area under the dose–response curves (AUC) was used as the summary statistics and subsequently utilized to rank the cell lines according to responsiveness. For each drug, cell lines were categorized as resistant (40% highest AUC), sensitive (40% lowest AUC), or intermediate (Figure 1a–d and Supplementary Table S3). Based on the proteomic profile of each cell line in untreated conditions, hierarchical clustering using Spearman correlation as the distance measure was performed, and the results are presented in the heatmap in Figure 1e. Hierarchical clustering of proteome profiles did not reveal distinct grouping of cell lines based on drug responses (resistant, intermediate, or sensitive) or cell-of-origin subtype (activated B-cell-like, germinal center B-cell-like, or unclassified) as indicated by the annotations in the figure.

### 3.2. Identification of Proteins Associated with Drug-Specific Response

**Figure 2:**
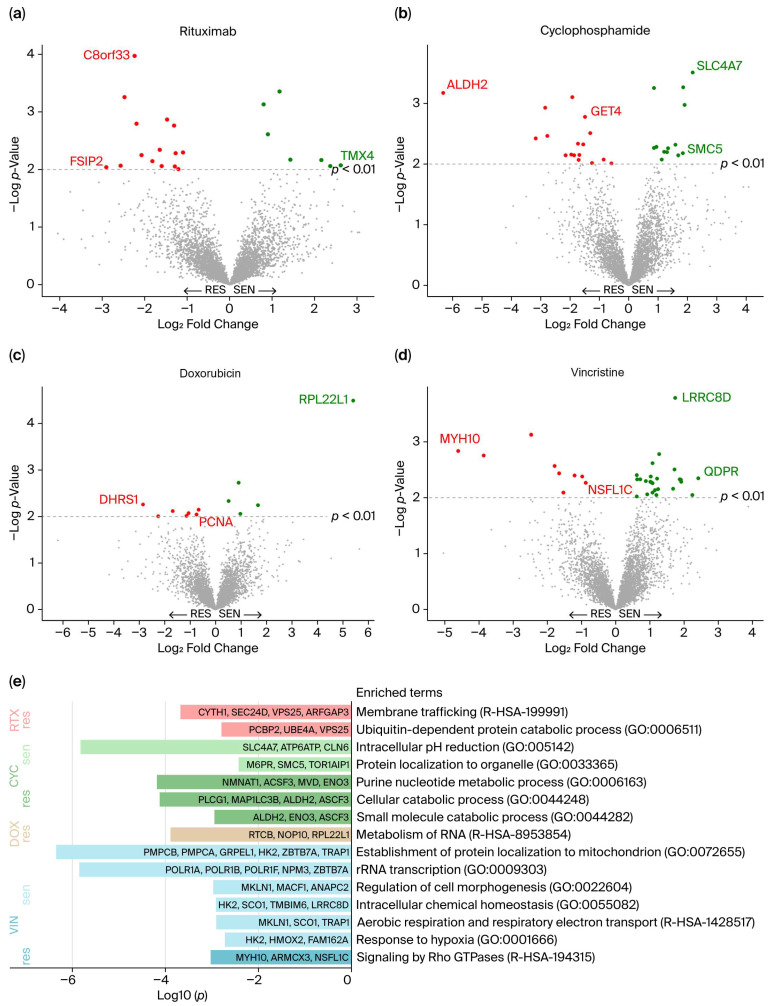
(**a**–**d**) Differential protein abundance between cell lines categorized as resistant and sensitive for each drug in R-CHO. The 98 significantly differentially abundant proteins (*p* < 0.01) are presented in red (more abundant in resistant cell lines) and green (more abundant in sensitive cell lines). The proteins with the greatest differences (Log_2_ Fold Change), the highest *p*-values, and those highlighted in Figure 3d–g are labeled. (**e**) Enriched biological pathways among the 98 differential proteins, presented with log10 (*p*-values). res, Resistant; sen, Sensitive; RTX, Rituximab; CYC, Cyclophosphamide; DOX, Doxorubicin; VIN, Vincristine.

To identify protein abundance levels associated with response to each drug in R-CHO, differential protein abundance analysis was performed between cell lines representing the extremes of drug responsiveness, i.e., sensitive vs. resistant. Cell lines categorized as intermediate were excluded from the analysis. In total, 98 differentially abundant proteins (*p* < 0.01) were identified between resistant and sensitive cell lines for all drugs (Figure 2a–d and Supplementary Table S4). For rituximab, 22 proteins showed differential abundance: 15 were more abundant in resistant cell lines, such as the proteins C8orf33 and FSIP2, and 7 proteins were more abundant in sensitive cell lines, such as TMX4. Comparison of cyclophosphamide sensitive and resistant cell lines revealed 30 differential proteins: 17 were highly abundant in resistant cell lines, including the proteins ALDH2 and GET4, and 13 were more abundant in sensitive cell lines, including SLC4A7 and SMC5. A total of 12 proteins were identified as differentially abundant between doxorubicin sensitive and resistant cell lines: 7 were more abundant in resistant cell lines (e.g., DHRS1 and PCNA), and 5 were more abundant in sensitive cell lines (e.g., RPL22L1). For vincristine, 9 proteins had higher abundance in resistant cell lines compared to sensitive cell lines, including the proteins MYH10 and NSFL1C, while 25 were more abundant in sensitive cell lines, such as LRRC8D and QDPR. No proteins were significantly differentially abundant for more than one drug. For simplicity, the proteins are referred to by their gene abbreviations.

### 3.3. Biological Functions of the Differentially Abundant Proteins

The 98 differentially abundant proteins were further examined for biological relevance. Pathway enrichment analyses were performed separately for proteins with high and low abundance in sensitive and resistant cell lines for each of the drugs. A total of 15 biological pathways were found to be enriched (Figure 2e). Pathway enrichment analysis revealed that highly abundant proteins in rituximab-resistant cell lines are involved in membrane trafficking and the ubiquitin-dependent protein catabolic process. For cells categorized by cyclophosphamide response, numerous pathways were enriched, notably the purine nucleotide metabolic process, including proteins enriched in resistant cells, such as NMNAT1, ACSF3, MVD and ENO3. This is especially interesting due to the pathway’s association with tumor growth [34]. Moreover, NAT1, whose overexpression is linked to carcinogenesis, especially breast cancer [35], was also highly abundant in cyclophosphamide-resistant cell lines. The two drugs with direct DNA-damaging effects, cyclophosphamide and doxorubicin [5], showed a substantial number of differentially abundant proteins involved in the DNA damage repair system (RNMT, SMC5, and VCPIP1 for cyclophosphamide, and PCNA, MMS19, and ASF1A for doxorubicin) [36,37]. Differentially abundant proteins related to doxorubicin response showed only one enriched term through pathway analysis, namely metabolism of RNA, which is a super pathway comprising a broad spectrum of functions. Vincristine-resistant cell lines showed increased abundance of proteins (MYH10, ARMCX3, and NSFL1C) involved in signaling by Rho GTPases. The Rho family of GTPases mainly regulates the actin cytoskeleton, thus supporting tumorigenesis through cancer cell growth, migration, and invasion [38]. Of the proteins involved in this pathway, NSFL1C is especially relevant, as it supports mitotic progression [39], which contradicts vincristine’s antimicrotubular mechanism that blocks mitosis.

### 3.4. Treatment-Associated Proteins in Cell Lines and Patient Samples

**Figure 3:**
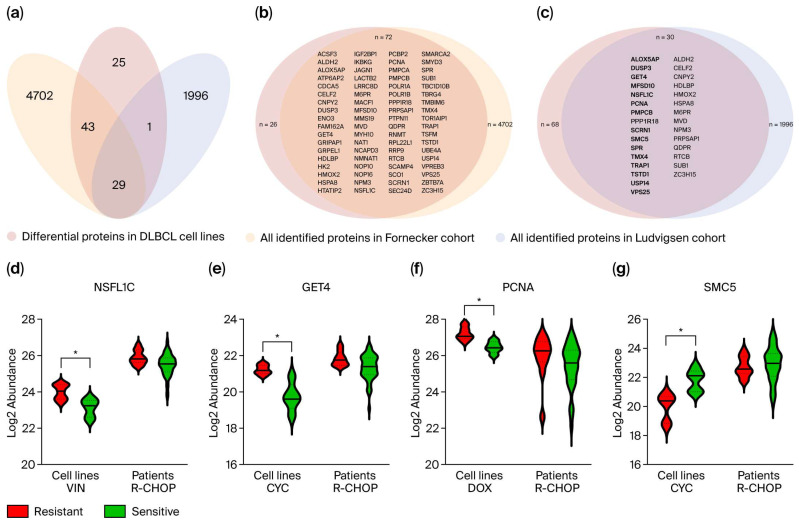
(**a**) The 98 differentially abundant proteins were examined for presence in two clinical cohorts, both including diagnostic DLBCL tumor tissue samples from patients with known subsequent R-CHOP treatment response. (**b**) Of the 98 differential proteins, 72 (73%) were present in the Fornecker cohort. (**c**) In the Ludvigsen cohort, 31% of the 98 differential proteins were identified, with 16 showing equivalent differential abundance patterns between resistant and sensitive groups in both cell lines and patient samples (highlighted in bold). (**d**–**g**) The log2 relative label-free quantitation (LFQ) values, that is, the protein abundance, of NSFL1C, GET4, PCNA, and SMC5 are shown for resistant and sensitive groups in cell lines and patient samples. Significant differences from the cell line analysis are shown with * for *p*-value < 0.01. VIN, Vincristine; CYC, Cyclophosphamide; DOX, Doxorubicin.

To determine whether the differentially abundant cell line proteins correspond to treatment response-associated proteins in clinical settings, their presence and abundance patterns were investigated in two clinical DLBCL proteomic profiling cohorts by Fornecker et al. [30] and Ludvigsen et al. [31]. Both cohorts included proteins identified in diagnostic DLBCL tumor tissue samples with known R-CHOP response (sensitive or resistant). Of the 98 differentially abundant proteins identified in the present DLBCL cell line proteomic study, 73% and 31% were also detected in the Fornecker and Ludvigsen cohorts, respectively (Figure 3a–c and Supplementary Table S5). Abundance values of these proteins in samples classified as resistant or sensitive were available from the Ludvigsen cohort, showing 16 proteins with similar differential abundant patterns between resistant and sensitive groups in cell lines and patient samples. These are shown in bold in Figure 3c, with their abundance patterns illustrated in Figure 3d–g and Supplementary Figure S1, and described in Supplementary Tables S5 and S6. Among these, GET4 and NSFL1C showed the highest enrichment (*p* < 0.05) in R-CHOP-resistant patient samples compared to sensitive ones. This aligns with our findings, where GET4 showed significantly higher abundance in cyclophosphamide-resistant cell lines, and NSFL1C was significantly enriched in vincristine-resistant cell lines (Figure 3d,e). These findings suggest that the higher abundance of GET4 and NSFL1C in R-CHOP-resistant patients can be attributed to single-drug components, that is, cyclophosphamide and vincristine, respectively. In the present DLBCL cell line study, NSFL1C and Rho GTPase signaling were shown to be associated with vincristine resistance. This is also in accordance with the findings in Ludvigsen et al., identifying members of the Rho family of GTPases enriched in R-CHOP-resistant patients. Two DNA damage repair-related proteins, SMC5 and PCNA, were found to be differentially abundant in cyclophosphamide- and doxorubicin-resistant and -sensitive cell lines, respectively. Both proteins also showed differential abundance in R-CHOP-resistant and -sensitive patients (Figure 3f,g).

## 4. Discussion

Proteomic studies of DLBCL have gained attention in the last decade. Several studies have focused on differential protein abundances between activated B-cell-like (ABC) and germinal center B-cell-like (GCB) subtypes [40,41,42,43,44]. These cell-of-origin subtypes have shown less impact on treatment outcomes than expected. Therefore, discovery proteomics investigating the landscape of proteins associated with treatment response has gained increasing attention [25,26,27]. However, to the authors’ knowledge, the current study is the first example of (a) single-drug R-CHOP-based analysis in a (b) comprehensive proteomics DLBCL cell line study. Previous studies have only conducted differential protein abundance analysis for the combined R-CHOP treatment. As this study reveals, this approach can obscure which proteins are associated with a specific single-drug response. This study, based on 16 DLBCL cell lines, contributes new proteomic data on 5 DLBCL cell lines with no previously published proteome information.

To investigate the proteome profiles of the 16 DLBCL cell lines, discovery-based proteomics using LC-MS/MS was performed, identifying 4716 proteins. Hierarchical clustering revealed no clear proteomic profile patterns to R-CHO responses, suggesting that R-CHO-associated proteins do not dominate the proteomic profiles. This is expected in such a comprehensive protein study encompassing a broad spectrum of variability, such as disease heterogeneity and different patient origins of the cell lines. Differential protein analysis between cell lines categorized as sensitive and resistant identified 98 proteins (*n* = 22 for rituximab, *n* = 30 for cyclophosphamide, *n* = 12 for doxorubicin, and *n* = 34 for vincristine), none of which overlapped among the drugs. This is most likely explained by the different mechanisms of action for the individual treatment components. Pathway analysis for rituximab-resistant cell lines showed enriched proteins (CYTH1, SEC24D, VPS25, and ARFGAP3) involved in membrane trafficking, which is a pathway mediating many processes and enzyme functions that cancer cells can use to promote tumorigenesis and drug responses [45]. Additionally, the ubiquitin-dependent protein catabolic process was enriched in rituximab-resistant cell lines. This is in concordance with the findings of Czuczman et al., which showed upregulation of components of the ubiquitin-proteasome system in rituximab-resistant cells. This was hypothesized to lead to degradation of the COOH-terminal of CD20 [46], suggesting a regulatory effect on CD20 expression. However, in the present study, the CD20 protein was not identified as differentially abundant between rituximab-resistant and -sensitive cell lines. Cyclophosphamide and doxorubicin exert their cytotoxicity by inducing DNA damage in cancer cells. Only for these two drugs, DNA damage repair-related proteins (ASF1A, MMS19, PCNA, RNMT, SMC5, and VCPIP1) were found to be differentially abundant between resistant and sensitive cell lines. This raises the question of the involvement of the DNA damage repair system in resistance mechanisms, especially against DNA-alkylating and -intercalating drugs, such as cyclophosphamide and doxorubicin. For vincristine-resistant cell lines, pathway analysis showed that three of the highly abundant proteins (MYH10, ARMCX3, and NSFL1C) are involved in Rho GTPase signaling. The large Rho family of GTPases is involved in diverse cellular processes, including regulation of the actin and microtubule cytoskeleton, as well as cell division [38,47]. These mechanisms support cancer cell growth, migration, and invasion [38]. More specifically, Rho GTPases are necessary for cell cycle progression, especially in G1/S phase transition [48] and during mitosis, where Rho GTPases are critical for spindle assembly [49]. This is opposite to vincristine’s mechanism of action, which inhibits the formation of mitotic spindles and blocks mitotic progression, revealing a drug-specific response mechanism [50]. Furthermore, upregulation of Rho GTPases and overactivation of their pathways have been associated with both tumorigenesis and tumor resistance [51,52]. This aligns with the enrichment of Rho GTPase signaling pathways in vincristine-resistant cell lines.

By examining two clinical DLBCL proteome cohorts, 72 and 30 proteins of the 98 differentially abundant cell line proteins were detected by Fornecker et al. [30] and Ludvigsen et al. [31], respectively. The lack of detection of some proteins in clinical samples might be caused by patient heterogeneity, including age, disease stage or subtype, comorbidities or other patient specific characteristics that obscure the protein detection. Other plausible explanations are the tumor microenvironment-related noise of the patient tissue samples or batch effects. The cohort in Ludvigsen et al. revealed considerably lower number of overlapping proteins compared to Fornecker et al. This may be due to the generally low protein detection in the cohort, with 2026 proteins identified in total versus 4774 in Fornecker et al. The lower detection rate in Ludvigsen et al. was possibly caused by formalin fixation of the samples and different filtration criteria, as proteins had to be present in at least 70% of the samples in each group. Some of the proteins showing the largest differential abundance in our cell lines study, such as C8orf33 (rituximab) and SLC4A7 (cyclophosphamide), were not detected in either of the cohorts, illustrating cell line model specificity. This minimizes their potential to be used as markers in clinical settings. Contrarily, proteins such as SPR (rituximab), GET4 (cyclophosphamide), and TSTD1 (vincristine) showed increased clinical potential when examined by Ludvigsen et al., as the proteins showed corresponding abundance patterns in the cell line and patient proteome datasets. This illustrates the importance of critically assessing pre-clinical model systems for relevance and broader representation of specific disease entities. Further examination of the abundance of proteins correspondingly identified by Ludvigsen et al. showed that 16 proteins exhibited the same differential abundance patterns between resistant and sensitive groups in both cell lines and patient samples. Many of these proteins have been linked to DLBCL and/or treatment responsiveness in other studies [53,54,55,56,57,58,59,60] (Supplementary Table S6), increasing their potential as DLBCL prognostic markers. The DNA damage repair-related protein PCNA showed a tendency to be more abundant in R-CHOP-resistant patient samples (Figure 3f) and was significantly more abundant in doxorubicin-resistant cell lines. This aligns with the findings of Mansoor et al., who reported upregulated *PCNA* gene expression in DLBCL and follicular lymphoma compared with normal tissue controls [56]. Moreover, PCNA has been proposed as a cancer therapeutic potential by targeting its DNA damage repair function to induce synthetic lethality [57]. This emphasizes the need for further examination of the effect of DNA damage repair proteins in both tumor development and treatment response in DLBCL. The significantly differentially abundant proteins between resistant and sensitive patients, GET4 and NSFL1C, are suggested to be associated with preparedness for response to cyclophosphamide and vincristine, respectively, in cell lines and clinical samples. NSFL1C exerts mitotic progressive function through fragmentation and reassembly of Golgi stacks during and after mitosis, regulating spindle orientation [39], and is involved in the enriched pathway “Signaling of Rho GTPases”. Hence, it is hypothesized that NSFL1C inhibits the effects of vincristine, leading to a resistant response, and thus encouraging further investigation. This is highly clinically relevant, as vincristine leads to severe neurotoxicity through microtubule disruptions, which is increased with higher doses [50]. Therefore, potential markers indicating a higher risk of vincristine resistance can suggest lower vincristine doses or substitute compounds for those patients. This approach can minimize the risk of adverse side effects while maintaining the maximum effectiveness of the standardized first-line R-CHOP regimen, which is important from a precision health perspective.

In conclusion, the identified proteins need further examination using more targeted protein analysis methods and larger clinical cohorts to elucidate their potential as companion diagnostic and prognostic markers for treatment. However, this study has identified their candidate potential.

## Data Availability

The mass spectrometry proteomics cell line data have been deposited in the ProteomeXchange Consortium via the PRIDE partner repository with the dataset identifier PXD064483.

## Supplementary Materials

The following supporting information can be downloaded at https://www.mdpi.com/article/10.3390/biomedicines13112709/s1.

## Abbreviations

The following abbreviations are used in this manuscript:

DLBCL	Diffuse large B-cell lymphoma
R-CHOP	Rituximab; cyclophosphamide; doxorubicin; vincristine; prednisone
rrDLBCL	Refractory or relapse DLBCL
CAR	Chimeric antigen receptor
ASCT	Autologous stem cell transplantation
LC-MS/MS	Liquid chromatography–mass spectrometry
AUC	Area under the curve
ABC	Activated B-cell-like
GCB	Germinal center B-cell-like
