# Supplementary material for: Proteomic Study of Diffuse Large B-Cell Lymphoma Identifying Proteins Associated with R-CHOP Response
Source: Biomedicines. 2025 Nov 4;13(11):2709. doi: 10.3390/biomedicines13112709 (PMC12650084; doi:10.3390/biomedicines13112709)
Supplement: Supplementary file 1 [file biomedicines-13-02709-s001.zip › Supplemental Figure S1 and Supplementa Table S6.pdf]

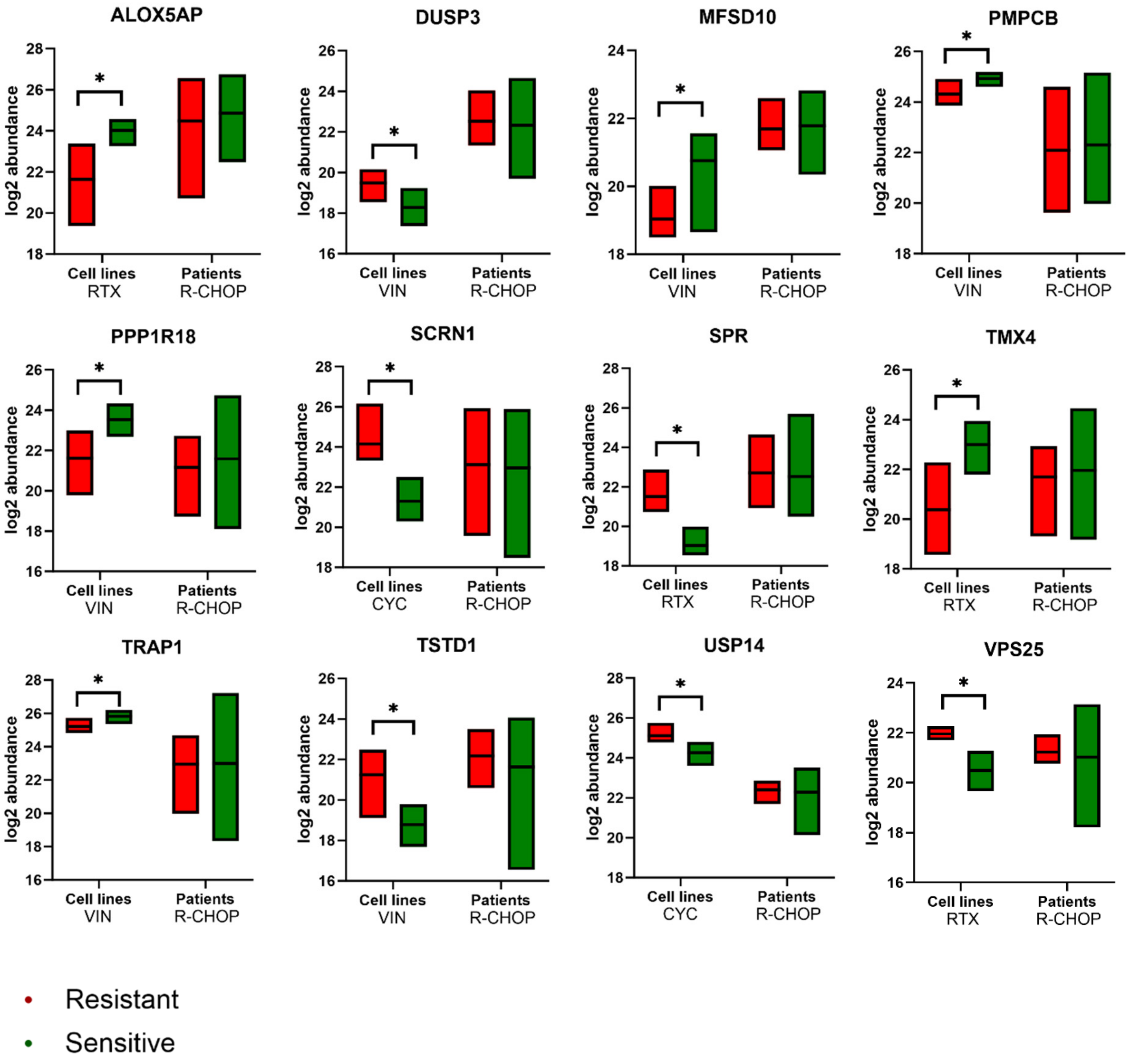

Supplementary Figure S1. The abundance of the 16 proteins with corresponding abundance patterns when comparing resistant and sensitive groups in DLBCL cell lines and patient samples. The log2 of the relative label free quantification is presented as protein abundance with mean of each group and significance (*=p<0.01) between resistant and sensitive groups.

Supplementary Table S6. Sen, sensitive; res, resistant; rtx, rituximab; cyc, cyclophosphamide; dox, doxorubicin; vin, vincristine

Proteins	Abundances	Functions (source: UniProt)	Associations in relevant studies
**ALOX5AP**	High in sen rtx	Important for leukotriene biosynthesis as it anchors ALOX5 to the cell membrane.	
**DUSP3**	High in res vin	Is a member of the dual specificity protein phosphatase subfamily which dephosphorylates proteins.	Has been found to have prognostic significance in DLBCL as high levels of DUSP3 significantly decreased overall survival of DLBCL patients [1].
**GET4**	High in res cyc	Is a component of the BAG6/BAT3 complex, a cytosolic protein quality control complex.	Is a driver gene in colorectal cancer [2].
**MFSD10**	High in sen vin	Is a member of the major facilitator superfamily of transporter proteins. Likely functions as organic anion transporter.	Is found to be hypermethylated and underexpressed in R/R DLBCL patients [3].
**NSFL1C**	High in res vin	Reduces the ATPase activity of valosin-containing protein (p97). Important for Golgi stacks fragmentation during mitosis and for p97-mediated reassembly of Golgi stacks after mitosis.	Significantly upregulated in R-CHOP resistant DLBCL patients [4] .
**PCNA**	High in res dox	Is a cofactor of DNA polymerase delta and in that way involved in eukaryotic DNA replication. This protein is ubiquitinated under DNA damage and involved in DNA repair through the RAD6-dependent pathway.	Found to be upregulated in DLBCL compared to FL and normal tissue controls [5]. Has been potentiated as a cancer therapeutic target through synthetic lethality[6].
**PMPCB**	High in sen vin	It catalyzes the cleavage of the mitochondrial sequence off newly imported precursor proteins.	
**PPP1R18**	High in sen vin	Protein phosphatase-1 interacts with regulatory subunits which target the enzyme to different cellular locations and change its activity toward specific substrates. May be a regulatory subunit that targets PP1 to F-actin cytoskeleton	
**SCRN1**	High in res cyc	Regulates exocytosis in mast cells.	

**SMC5**	High in sen cyc	Involved in the homologous recombination DNA damage repair pathway as a component of the SMC5-SMC6 complex.	
**SPR**	High in res rtx	An aldo-keto reductase which catalyzes the NADPH-dependent reduction of pteridine derivatives. Involved in the biosynthesis of tetrahydrobiopterin (BH4).	
**TMX4**	High in sen rtx	Is a member of the disulfide isomerase (PDI) family of endoplasmic reticulum (ER) proteins. These catalyze protein folding and thiol-disulfide interchange reactions.	
**TRAP1**	High in sen vin	A mitochondrial chaperone protein and a member of the heat shock protein 90 (HSP90) family. It has ATPase activity and interacts with tumor necrosis factor type I. Probable function as cellular stress responses regulator.	
**TSTD1**	High in res vin	May to enable thiosulfate-thiol sulfurtransferase activity. Probable involvement in sulfide oxidation, using sulfide:quinone oxidoreductase.	Higher expression have been correlated to poor chemotherapeutic response (also alkylating drugs)[8].
**USP14**	High in res cyc	A member of the ubiquitin-specific processing (UBP) family of proteases that is a deubiquitinating enzyme (DUB) with His and Cys domains. Located in the cytoplasm and cleaves the ubiquitin moiety from ubiquitin-fused precursors and ubiquitinylated proteins.	Has been shown to be highly expressed in DLBCL and having potential as therapeutic target [9].
**VPS25**	High in res rtx	This gene encodes a protein that is a subunit of the endosomal sorting complex required for transport II (ESCRT-II). This protein complex functions in sorting of ubiquitinated membrane proteins during endocytosis.	

[1] Fang RR, Xu Y, Qi C, Morse H, Zhou JX. The Prognostic Significance Of DUSP3 In DLBCL. Blood 2013;122:3025–3025. https://doi.org/10.1182/blood.v122.21.3025.3025.

[2] Koike K, Masuda T, Sato K, Fujii A, Wakiyama H, Tobo T, et al. GET4 is a novel driver gene in colorectal cancer that regulates the localization of BAG6, a nucleocytoplasmic shuttling protein. Cancer Sci 2022;113:156–69. https://doi.org/10.1111/cas.15174.

[3] Kendall EK, Patel MS, Ondrejka S, Mian A, Sawalha Y, Hu B, et al. Integrative DNA Methylation and Gene Expression Analysis Reveals Candidate Biomarkers Associated with Dichotomized

Response to Chemoimmunotherapy in Diffuse Large B-Cell Lymphoma. Blood 2020;136:22–22. https://doi.org/10.1182/blood-2020-137180.

[4] Ludvigsen M, Campbell AJ, Enemark MB, Hybel TE, Karjalainen-Lindsberg ML, Beiske K, et al. Proteomics uncovers molecular features for relapse risk stratification in patients with diffuse large B-cell lymphoma. Blood Cancer J 2023;13:11–4. https://doi.org/10.1038/s41408-023-00931-6.

[5] Mansoor A, Kamran H, Rizwan H, Akhter A, Roshan TM, Shabani-Rad MT, et al. Expression of “DNA damage response” pathway genes in diffuse large B-cell lymphoma: The potential for exploiting synthetic lethality. Hematol Oncol 2024;42:1–10. https://doi.org/10.1002/hon.3225.

[6] Li CM, Haratipour P, Lingeman RG, Perry JJP, Gu L, Hickey RJ, et al. Novel peptide therapeutic approaches for cancer treatment. Cells 2021;10:2908. https://doi.org/10.3390/cells10112908.

[7] Wong KK, Gascoyne DM, Soilleux EJ, Lyne L, Spearman H, Roncador G, et al. FOXP2-positive diffuse large B-cell lymphomas exhibit a poor response to R-CHOP therapy and distinct biological signatures. Oncotarget 2016;7:52940–56. https://doi.org/10.18632/oncotarget.9507.

[8] Ansar M, Thu LTA, Hung CS, Su CM, Huang MH, Liao LM, et al. Promoter hypomethylation and overexpression of TSTD1 mediate poor treatment response in breast cancer. Front Oncol 2022;12. https://doi.org/10.3389/fonc.2022.1004261.

[9] Jiang L, Sun Y, Wang J, He Q, Chen X, Lan X, et al. Proteasomal cysteine deubiquitinase inhibitor b-AP15 suppresses migration and induces apoptosis in diffuse large B cell lymphoma. J Exp Clin Cancer Res 2019;38:453. https://doi.org/10.1186/s13046-019-1446-y.
